# Impact of BNT162b2 mRNA Vaccination on the Development of Short and Long-Term Vaccine-Related Adverse Events in Inflammatory Bowel Disease: A Multi-Center Prospective Study

**DOI:** 10.3389/fmed.2022.881027

**Published:** 2022-06-08

**Authors:** Mohammad Shehab, Fatema Alrashed, Israa Abdullah, Ahmad Alfadhli, Hamad Ali, Mohamed Abu-Farha, Arshad Mohamed Channanath, Jehad Ahmed Abubaker, Fahd Al-Mulla

**Affiliations:** ^1^Division of Gastroenterology, Department of Internal Medicine, Mubarak Alkabeer University Hospital, Kuwait University, Jabriya, Kuwait; ^2^Department of Pharmacy Practice, Faculty of Pharmacy, Health Sciences Center (HSC), Kuwait University, Jabriya, Kuwait; ^3^Clinical Pharmacy Unit, Department of Pharmacy, Kuwait Hospital, Sabah Al-Salem, Kuwait; ^4^Department of Genetics and Bioinformatics, Dasman Diabetes Institute (DDI), Kuwait City, Kuwait; ^5^Department of Medical Laboratory Sciences, Faculty of Allied Health Sciences, Health Sciences Center (HSC), Kuwait University, Kuwait City, Kuwait; ^6^Department of Biochemistry and Molecular Biology, Dasman Diabetes Institute (DDI), Kuwait City, Kuwait

**Keywords:** IBD, COVID-19, vaccine, safety, symptoms

## Abstract

**Introduction:**

Severe acute respiratory syndrome coronavirus 2 (SARS-CoV-2) vaccination has been effective in protecting against severe COVID-19 infections and related mortality. It is recommended for all individuals including patients with inflammatory bowel disease (IBD). However, safety data are lacking in this group of patients. Therefore, we aim to evaluate the short- and long-term vaccine related adverse events (AEs) in patients with IBD.

**Methods:**

This is a prospective, observational cohort study investigating short- and long-term AEs related to the BNT162b2 vaccine in patients with IBD (study group) after the first and second dose compared to healthy participants (control group). Patients were recruited at the time of attendance to the clinic or infusion rooms. Short term (<3 weeks) localized and systemic AEs were assessed *via* questionnaire. Follow-up phone-based survey was made to collect data on long term (up to 24 weeks) AEs.

**Results:**

A total of 408 patients answered the questionnaires, 204 patients in each group, the study and control group. No serious adverse events were reported in either the study or the control group after the first or the second dose. Participants in the control group reported more frequent pain at the injection site than those in the study group after the first dose [58 (57%) vs. 38 (37%) respectively, *P* = 0.005]. After the second dose, tiredness was reported more frequently in the control group [49 (48%)] compared to the study group [25 (24%) (*P* < 0.001)]. At 20–24 weeks post vaccination, 386 out of 408 (94.6%) patients were willing to participate in the follow-up phone based questionnaire [196 (96.1%) in the study group vs. 190 (93.1%) in the control group]. In both groups, none of the patients reported local, systemic, or severe adverse events (0 out of 386) at week 20–24 post second dose.

**Conclusion:**

The BNT162b2 vaccine is safe in patients with IBD. No severe or long-term adverse events were reported in our study. The frequency of local and systemic adverse events after the second dose was generally higher among healthy participants compared to patients with IBD. Further studies including a larger cohort with a longer follow-up duration are needed to assess for possible rare adverse events.

## Introduction

The severe acute respiratory syndrome coronavirus 2 (SARS-CoV-2) emerged in Wuhan, China in December 2019 (1). The disease has been known to cause a significant morbidity and mortality among many of those infected. The outbreak, which was later declared a pandemic, had global health and socioeconomic consequences (2). This has led to an international effort for vaccine development and the introduction of the first vaccine, the BNT162b2 mRNA (Pfizer/BioNTech) vaccine in December 2020 followed by ChAdOx1 nCoV-19, mRNA-1273 (Moderna) and other vaccines, which later were authorized under the emergency use authorization (3, 4).

Thereafter, clinical trials and real-world data have shown efficacy and safety of these vaccines in reducing COVID-19 infection severity and decreasing both hospitalization and mortality in patients with COVID-19. Nevertheless, patients with inflammatory bowel disease (IBD) were largely excluded from these trials (5–7).

Despite the fact that many of the patients with IBD are on immune-modifying medications, those patients were not found to be at higher risk of developing COVID-19 infection. However, being on corticosteroids was found to be a risk factor for developing more severe infection (8). Vaccination against the SARS-CoV-2 virus in patients with IBD is highly recommended by most international gastrointestinal societies (9, 10).

A study showed that the overall prevalence of COVID-19 vaccination among patients with IBD on biologic therapies was lower than that of the general population (11). Furthermore, many studies focused on the efficacy of COVID-19 vaccination in patients with IBD receiving biologic therapies, while the safety of vaccination was not extensively explored (12, 13). However, evidence regarding the vaccine safety in patients with IBD is slowly emerging, with the majority of studies investigating only the short-term adverse events following vaccination in patients with IBD (14, 15). Therefore, it is imperative to assess the long-term safety of SARS-CoV-2 vaccine in patients with IBD. This study aims to evaluate the short- and long-term adverse events following vaccination with BNT162b2 mRNA vaccine among patients with IBD.

## Materials and Methods

We performed a prospective multi-center cohort study at two tertiary care centers (Muabark Alkabeer Hospital and Dasman Center) to assess short and long-term adverse events related to COVID-19 mRNA vaccine, BNT162b2 (Pfizer/BioNTech) in patients with IBD (study group) compared to healthy participants (control group).

This study was performed and reported in accordance with Strengthening the Reporting of Observational Studies in Epidemiology (STROBE) guidelines (16). This study was reviewed and approved by the Ethical Review Board of Mubarak Alkabeer Hospital and Dasman Center “Protocol # RA HM-2021-008” as per the updated guidelines of the Declaration of Helsinki (64th WMA General Assembly, Fortaleza, Brazil, October 2013) and of the US Federal Policy for the Protection of Human Subjects. The study was also approved by the regional health authority (reference: 3799, protocol number 1729/2021). Subsequently, patient informed written consent was obtained before inclusion in the study.

Localized and systemic adverse events to the BNT162b2 vaccine were assessed *via* paper questionnaires at the time of attendance at the gastroenterology infusion rooms and outpatient clinics from 1 August 2021 to 15 September 2021, and patients were followed up to 10 February 2022, using a phone based questionnaire. Outcomes were stratified by first and second doses.

Study group patients were eligible to be included if they: (1) had confirmed diagnosis of inflammatory bowel disease (IBD) before the start of the study, (2) had received one or two doses of COVID-19 vaccination with BNT162b2 (Pfizer-BioNTech), (3) were at least 18 years of age or older. Patients were excluded if they received any vaccine other than the BNT162b2 or if they tested positive for SARS-CoV-2 previously or had symptoms of COVID-19 since the start of the pandemic up to the time of vaccination. Patients were also excluded if they have one of the following within 8 weeks of vaccination: stool fecal calprotectin levels >250 ug/g, C-Reactive Protein (CRP) levels >10 mg/L, active symptoms of IBD or endoscopic active disease (refer below), use of corticosteroids or active extraintestinal manifestation of IBD (e.g., inflammatory uveitis, arthritis, skin rashes, etc).

Patients with Harvey Bradshaw Index (HBI) >4 and partial clinical Mayo score >1 are considered to have active symptoms of IBD. In addition, patients who had colonoscopies with an endoscopic Mayo score >1 for ulcerative colitis or Simple Endoscopic Score for Crohn's Disease (SES-CD) >4 are considered to have active endoscopic. In addition, patients who had severe allergic reactions to a previous vaccine in their life or were unwilling to participate in the study were excluded.

On the other hand, healthy participants (control) group were individuals who volunteered to participate in the study at Dasman Center with no previous history of chronic medical illnesses such as diabetes, hypertension, cardiovascular disease, autoimmune diseases, osteoarthritis, chronic obstructive pulmonary disease, renal disease, asthma, hyperlipidemia, or history of stroke and bleeding disorder. In addition, basic laboratory tests were performed (full blood count, renal function tests, liver function tests, lipid profile, HbA1c, ESR, and CRP) to objectively screen for underlying diseases.

The baseline questionnaire assessed the type of immunization, date of immunization(s), patient demographics and IBD characteristics, and data regarding IBD medication use around the time of vaccination. Participants were asked to report short-term localized and systemic adverse events defined as adverse events occurring within 14 days after receiving the BNT162b2 vaccine dose 1 and 21 days of receiving vaccine dose 2. The follow-up phone-based survey collected data on long term (20–24 weeks from the first dose) adverse events of BNT162b2 vaccination. Both groups were tested with SARS-CoV-2 PCR within 72 h before each vaccine dose. Positive subjects were excluded from the study. Patients were also monitored 24 weeks post vaccination. Any subjects who reported symptoms of COVID-19 or tested positive for it were also excluded.

Vaccine adverse events were classified as the injection site (localized) or systemic reactions. Adverse localized reactions included pain, redness, itching, swelling, or tenderness at the injection site. Systemic adverse reactions included fever, chills, fatigue, headache, joint pain, muscle aches, nausea, allergic reaction, rash, or other. Severe adverse events were defined as incidence of pulmonary embolism, acute myocardial infarction, immune thrombocytopenia, and disseminated intravascular coagulation per the Food and Drug Administration (FDA) definition of potential adverse events of interest (17).

Diagnosis of inflammatory bowel disease (IBD) was made according to the international classification of diseases (ICD-10 version: 2016). Patients were considered to have IBD when they had ICD-10 K50, K50.1, K50.8, K50.9 corresponding to Crohn's disease (CD) and ICD-10 K51, K51.0, K51.2, K51.3, K51.5, K51.8, K51.9 corresponds to ulcerative colitis (UC) (18).

### Statistical Analysis

Analysis was conducted using R (19). We performed descriptive statistics to present the demographic characteristics of patients included in this study. The McNemar test was used to determine whether the proportion of participants who had any symptoms (yes or no) after the first dose of the vaccine differed after the second dose. Pearson's Chi-squared test was used to compare the proportions of symptoms of the control group vs. the study group. Participants in both groups were matched for *Age* and *Gender*. The technique attempts to choose matches that collectively optimize an overall criterion. The criterion used is the sum of the absolute pair distances in the matched sample.

## Results

### Baseline Characteristics

Between 1 August 2021 and 15 September 2021, a total of 204 patients diagnosed with inflammatory bowel disease (IBD) answered the questionnaire. Of these, 119 (58%) were males. The median age of the patients included was 34.6 years (IQR 25–41). A total of 140 (68.7%) patients and 64 (31.3%) patients had Crohn's disease and ulcerative colitis, respectively. Most patients were receiving biologic therapy [82(40%)], followed by immunomodulators [75 (37.0%)], whereas 47 (23.0%) patients were on 5-aminosalicylates.

Before receiving the first dose, the mean CRP was 7 mg/L in the study group and 5 mg/L in the control group. Mean stool fecal calprotectin was 85 mcg/g in the study group.

In the study group, half of the patients (*n* = 102) received one dose of the Pfizer-BioNTech vaccine, while the other half (*n* = 102) received two doses of the vaccine. Asthma (8%), arthritis (5%), and diabetes (3.9%) were the most common comorbidities in the study group. Demographics are shown in Table 1. No serious adverse events were reported in either the study or the control group after the first or the second dose.

**Table 1:** Baseline characteristics of patients with inflammatory bowel disease (IBD).

**Characteristic**	***N* = 204**
**Age (years)** ^a^	34.6 (25, 41)
**Gender**	
Female	85 (42%)
Male	119 (58%)
**Nationality**	
Kuwaiti	180 (88%)
Non-Kuwaiti	24 (12%)
**Disease extent**, ***n*** **(%)**	
Ulcerative colitis (UC)	64 (31.3%)
E1: ulcerative proctitis	12 (18.7%)
E2: left sided colitis	20 (31.2%)
E3: extensive colitis	32 (50.0%)
Crohn's disease (CD)	140 (68.7%)
L1: ileal	62 (44.2%)
L2: colonic	24 (17.1%)
L3: ileocolonic	48 (34.4%)
L4: upper gastrointestinal	6 (4.3%)
B1: inflammatory	68 (48.8%)
B2: structuring	33 (23.3%)
B3: penetrating	39 (27.7%)
**Past medical history**	
Diabetes	8 (3.9%)
Hypertension	5 (2.0%)
Heart disease	5 (2.0%)
Arthritis	11 (5.0%)
COPD	2 (1.0%)
Kidney	5 (2.0%)
Asthma	16 (8.0%)
Hyperlipidemia	7 (3.0%)
**IBD medications**	
5-aminosalicylates	47 (23.0%)
Immunomodulators	75 (37.0%)
Biologics and small molecules	82 (40.0%)

a *Median (IQR) or frequency (%)*.

### Symptoms After the First Dose in the Study Group vs. Control Group

Adverse events after the first vaccine dose are shown in Table 2. The most common local adverse event was pain at the injection site reported in 37% of the study group. In general, after receiving the first dose, local adverse events were reported more frequently by patients in the control group. Specifically, subjects in the control group reported more frequent pain at the injection site than those in the study group [58 (57%) vs. 38 (37%), respectively, *P* = 0.005]. Redness and swelling at the injection site were also more common in the control group compared to the study group, [17 (17%) vs. 3 (2.9%), *P* < 0.001, and 18 (18%) vs. 4 (3.9%), *P* = 0.002], respectively.

**Table 2:** Comparison of symptoms after the first dose in patients with IBD (study) group vs. healthy participants (control) group.

**Characteristic**	**Study group, *N* = 102** ^a^	**Control group, *N* = 102** ^a^	***P* ^b^**
**Age_in years**	33 (26, 41)	34 (28, 43)	
**Gender**			
Female	48 (47%)	52 (51%)	
Male	54 (53%)	50 (49%)	
**Injection site**			
Pain	38 (37%)	58 (57%)	0.005
Redness	3 (2.9%)	17 (17%)	<0.001
Swelling	4 (3.9%)	18 (18%)	0.002
**Systemic AEs**			
Tiredness	19 (19%)	35 (34%)	0.011
Headache	15 (15%)	16 (16%)	0.8
Muscle pain	11 (11%)	24 (24%)	0.016
Chills	4 (3.9%)	14 (14%)	0.014
Fever	13 (13%)	14 (14%)	0.8
Nausea	1 (1.0%)	9 (8.8%)	0.009
Joint pain	8 (7.8%)	11 (11%)	0.5

a *Median (IQR) or frequency (%)*.

b *Wilcoxon rank sum test; Pearson's Chi-squared test*.

Similarly, after receiving the first dose, more subjects reported systemic reactions in the control group compared to the study group. Specifically, tiredness was reported by 35 (34%) of the subjects in the control group as opposed to 19 (19%) subjects in the study group (*P* = 0.011), muscle pain in 24 (24%) subjects in the control group and 11 (11%) subjects in the study group (*P* = 0.016), chills in 14 (14%) subjects in the control group compared to 4 (3.9%) subjects in the study group (*P* = 0.014), and nausea in 9 (8.8%) subjects from the control group compared to 1 (1.0%) subjects in the study group (*P* = 0.009). There was no significant difference in the occurrence of headaches, fever, or joint pain between the control group and the study group after the first dose of the vaccine.

### Symptoms After the Second Dose in the Study Group vs. Control Group

The frequency of local adverse events after the second dose was also generally higher among subjects in the control group than those in the study group (Table 3). Pain at the injection site was reported by 50 (49%) subjects in the control group and 32 (31%) subjects in the study group (*P* = 0.01). Additionally, 15 (15%) subjects in the control group reported redness at the injection site compared to 4 (3.9%) subjects in the study group (*P* = 0.008), while 29 (28%) subjects had swelling at the injection site in the control group compared to 3 (2.9%) subjects in the study group (*P* < 0.001).

**Table 3:** Comparison of symptoms after the second dose in patients with IBD (study) group vs. healthy participants (control) group.

**Characteristic**	**Study group, *N* = 102** ^a^	**Control group, *N* = 102** ^a^	***P* ^b^**
**Age_in years**	34 (25, 41)	33 (25, 45)	
**Gender**			
Female	43 (42%)	49 (48%)	
Male	59 (58%)	53 (52%)	
**Injection site**			
Pain	32 (31%)	50 (49%)	0.010
Redness	4 (3.9%)	15 (15%)	0.008
Swelling	3 (2.9%)	29 (28%)	<0.001
**Systemic AEs**			
Tiredness	25 (24%)	49 (48%)	<0.001
Headache	21 (20%)	35 (34%)	0.001
Muscle pain	16 (15%)	34 (33%)	<0.001
Chills	9 (8.8%)	19 (19%)	0.042
Fever	20 (20%)	26 (25%)	0.3
Nausea	2 (2.0%)	14 (14%)	0.002
Joint pain	4 (3.9%)	20 (20%)	<0.001

a *Median (IQR) or frequency (%)*.

b *Wilcoxon rank sum test; Pearson's Chi-squared test*.

When comparing systemic reactions among subjects in the control group as opposed to the study group, tiredness was reported by 49 (48%) subjects vs. 25 (24%) subjects (*P* < 0.001), headaches in 35 (34%) subjects vs. 21 (20%) (*P* = 0.001), muscle pain in 34 (33%) vs. 16 (15%) subjects (*P* < 0.001), chills in 19 (19%) vs. 9 (8.8%) subjects (*P* = 0.042), nausea in 14 (14%) subjects vs. 2 (2.0%) subjects (*P* = 0.002), and joint pain in 20 (20%) vs. 4 (3.9%) subjects (*P* < 0.001) respectively. Conversely, no significant difference was found in the frequency of fever between the control group and the study group [26 (25%) vs. 20 (20%), *P* = 0.3] (Table 3).

Other than nausea, none of the participants reported gastrointestinal (GI) related symptoms such as diarrhea or abdominal pain after the first or second dose. Furthermore, none of the patients reported any severe adverse events after the first or second dose. Additionally, no significant differences in any adverse reaction frequency were seen based on sex or age.

### Symptoms After the First Dose vs. the Second Dose in the Study Group

The frequency and type of adverse reactions after the first dose of the vaccine were compared with adverse reactions after the second dose of the vaccine among patients with IBD (study group). There was no significant difference in the frequency of symptoms reported after the first and second dose among subjects in the study group. The most common local reaction was pain at the injection site reported by 38 (37%) patients after the first dose and 32 (31%) patients after the second dose (*P* = 0.3). Redness was reported in 3 (2.9%) subjects after the first dose and 4 (3.9%) after the second dose (*P* > 0.9) and swelling 4 (3.9%) subjects after the first dose and in 3 (2.9%) after the second dose (*P* > 0.9).

Systemic reactions were reported as follows: tiredness in 19 (19%) after dose 1 vs. 25 (24%) after dose 2, *P* > 0.9, headache in 15 (15%) after dose 1 vs. 21 (20%) after dose 2, *P* > 0.9, muscle pain in 11 11% after dose 1 vs. 16 (15%) after dose 2, *P* > 0.9, chills in 4 (3.9%) after dose 1 vs. 9 (8.8%) after dose 2, *P* = 0.2, fever in 13 (13%) after dose 1 vs. 20 (20%) after dose 2, *P* = 0.2, nausea in 1 (1%) after dose 1 vs. 2 (2%) after dose 2, *P* > 0.9, and joint pain in 8 (7.8%) after dose 1 vs. 4 (3.9%) after dose 2, *P* = 0.3 (Table 4).

**Table 4:** Symptoms after the first dose and second dose in patients with IBD (study group).

**Characteristic**	**First dose, *N* = 102^a^**	**Second dose, *N* = 102^a^**	***P* ^b^**
**Injection site**			
Pain	38 (37%)	32 (31%)	0.3
Redness	3 (2.9%)	4 (3.9%)	>0.9
Swelling	4 (3.9%)	3 (2.9%)	>0.9
**Systemic AEs**			
Tiredness	19 (19%)	25 (24%)	>0.9
Headache	15 (15%)	21 (20%)	>0.9
Muscle pain	11 (11%)	16 (15%)	>0.9
Chills	4 (3.9%)	9 (8.8%)	0.2
Fever	13 (13%)	20 (20%)	0.2
Nausea	1 (1.0%)	2 (2.0%)	>0.9
Joint pain	8 (7.8%)	4 (3.9%)	0.3

a *n (%)*.

b *McNemar's Chi-squared test with continuity correction; McNemar's Chi-squared test*.

### Long-Term Adverse Events

At 20–24 weeks post vaccination, 386 out of 408 patients were willing to participate in the follow-up phone-based questionnaire. In the study group, 196 (96.1%) patients and 190 (93.1%) in the control group answered the questionnaire. 21 (10.7%) out of 196 patients in the study group, and 19 (10.0%) out of 190 in the control group reported having a breakthrough SARS-CoV-2 infection confirmed by PCR test after the second dose of vaccination. None of the patients who tested positive were hospitalized.

In both groups, none of the patients reported local, systemic, or severe adverse events (0 out of 386). In addition, short-term local and systemic adverse events have been resolved in the control and study groups.

## Discussion

We performed a survey-based study to explore the onset of adverse events related to the BNT162b2 vaccine in patients with inflammatory bowel disease (IBD) compared with healthy participants. In our study, none of our patients had severe vaccine-related adverse events, as they are very rare (20). We found that the most common adverse events after the first and the second doses were tiredness and headache, followed by local pain at the injection site. Nausea was reported in both study and control group, however, none of the groups reported other gastrointestinal (GI) related symptoms such as diarrhea or abdominal pain.

Weaver et al. (21) explored vaccine related adverse events among patients with IBD and the effect of vaccination on IBD disease course. Similar to our study, they found that severe localized and systemic vaccine-related adverse events were rare in patients with IBD. Injection site tenderness (68%) and fatigue (46% dose 1, 68% dose 2) were the most commonly reported localized and systemic adverse events after vaccination.

Interestingly, we also found that local and systemic adverse events were more common in healthy participants compared to patients with IBD. Given that the majority of our IBD cohort are on biologics or immunomodulators, it is possible that these medications blunt the immune response to vaccination. Botwin et al. (14) evaluated post-mRNA vaccination adverse events in 246 vaccinated adults with IBD participating in a longitudinal vaccine registry. Similar to our finding, the study found that adverse events were less common in individuals receiving biologic therapy. The authors concluded that patients with IBD can be reassured that the risk of adverse events is likely not increased, and may be reduced while receiving concomitant biologic therapy.

None of the participants in our study experienced severe short- or long-term adverse events. One systematic review and meta-analysis evaluated SARSCoV-2 vaccination in patients with IBD. The study did not find any severe adverse events or vaccine-related mortality in patients with IBD and the majority of patients reported mild adverse events after vaccination, including fatigue, headache, dizziness, and gastrointestinal symptoms (22).

Another study (23) observed a higher rate of diarrhea and abdominal pain in vaccinated patients with IBD compared to the general population. They also found that age and disease remission was inversely correlated with the onset of GI symptoms. To our knowledge, no evidence has emerged of IBD flare-ups caused by COVID-19 vaccination. Furthermore, in a population-based study, the effect of SARS-CoV-2 vaccination on IBD course was evaluated for a period of 4 weeks in patients with IBD. The study reported no clinical and laboratory exacerbation compared with the pre-vaccination baseline and no increase in corticosteroid prescription 1 month after vaccination in a large retrospective cohort compared with a matched unvaccinated cohort (24).

Another study (15) explored immediate (within 1 day) adverse events after mRNA SARS-CoV-2 vaccines in patients with IBD in the United States. Similar to our study, the authors found that the incidence of adverse events including acute myocardial infarction, anaphylaxis, facial nerve palsy, and coagulopathy in patients with IBD after COVID-19 vaccination was small and similar to a matched cohort of patients without IBD. Immediate adverse events after vaccination were rare in both cohorts.

In our study, none of our patients reported long-term adverse events 20–24 weeks after vaccination. One study (20) involved more than 1.5 million BNT162b2 vaccinated persons from an integrated healthcare organization, followed over a period of 42 days. The study reported an excess risk of lymphadenopathy (78.4 events per 1,00,000 persons), herpes zoster infection (15.8 events), appendicitis (5.0 events), and myocarditis (2.7 events) in the vaccinated cohort. However, the author concluded that their results indicate that SARS-CoV-2 infection is itself a very strong risk factor for myocarditis, and it also substantially increases the risk of multiple other serious adverse events.

Taken together, these emerging data provide reassurance that COVID-19 vaccination does not cause severe adverse events in patients with IBD and support recent consensus recommendations to vaccinate all patients with IBD. British Society of Gastroenterology (BSG) (10), the International Organization for the Study of Inflammatory Bowel Diseases (IOIBD) (25), and the Canadian Association of Gastroenterology (9) recommend that all patients with IBD should receive SARS-CoV-2 vaccination regardless of whether patients were in remission or not. In addition, the CORALE-IBD study group is assessing and conducting studies at Cedars-Sinai to understand the effects of vaccination against COVID-19 in people with IBD, and their recent publications reassured patients with IBD and provider communities that symptoms after the second and third dose of mRNA vaccine are generally mild and well tolerated. It also showed that responses after mRNA vaccination in adults with IBD receiving various medication regimens are robust (26, 27).

Our study has several strengths. It provides real world data for the public about adverse effects and vaccine safety in a subpopulation that was not studied in the initial clinical trials. In addition, the comparison to healthy participants and the rigorous matching allowed for precise estimation of the rate of adverse events in patients with IBD. Finally, the long-term follow-up period helps detect any possible late events that may occur several weeks after vaccination.

Our study also has some limitations. Given the observational nature of this study, it is possible that some hidden confounding variables were still not properly addressed. In addition, this study was performed prospectively in the context of an ongoing pandemic, therefore, the association between any breakthrough infection and any given adverse events cannot be ruled out. In addition, COVID antibody testing was not performed for all patients which could help identify those patients with previous silent infection. However, in our follow-up phone-based survey we asked patients about any previous or current SARS-CoV-2 infection. Despite these limitations, our study provides highly anticipated data regarding the short- and long-term adverse events of the SARS-CoV-2 vaccination in patients with IBD.

## Conclusion

The BNT162b2 vaccine is safe in patients with IBD. No severe or long-term adverse events were reported in our study. The frequency of local and systemic adverse events after the second dose was generally higher among healthy participants compared to patients with IBD. Further studies including a larger cohort with a longer follow-up duration are needed to assess for possible rare adverse events.

## Data Availability Statement

The data presented in this study are available upon request from the corresponding author. The data are not publicly available due to local legal and ethical regulations.

## Ethics Statement

This study was reviewed and approved by the Ethical Review Board of Mubarak Alkabeer Hospital and Dasman Center Protocol # RA HM-2021-008 as per the updated guidelines of the Declaration of Helsinki (64th WMA General Assembly, Fortaleza, Brazil, October 2013) and of the US Federal Policy for the Protection of Human Subjects. The study was also approved by the Regional Health Authority (Reference: 3799, Protocol Number 1729/2021). Subsequently, the patient informed written consent was obtained before inclusion in the study. The patients/participants provided their written informed consent to participate in this study.

## Author Contributions

MS: study concept and design, acquisition of data, analysis and interpretation of data, drafting of the manuscript, critical revision of the manuscript for important intellectual content, and submission of the manuscript. FA, MS, and IA: acquisition of data and drafting of the manuscript. AC: statistical analysis and interpretation of data. HA: data collection and supervision. JA and FA: critical revision of the manuscript for important intellectual content and study supervision. All the authors contributed to the article and approved the submitted version.

## Funding

This study was funded by Kuwait Foundation for the Advancement of Sciences (KFAS) Grant (RA HM-2021-008).

## Conflict of Interest

The authors declare that the research was conducted in the absence of any commercial or financial relationships that could be construed as a potential conflict of interest.

## Publisher's Note

All claims expressed in this article are solely those of the authors and do not necessarily represent those of their affiliated organizations, or those of the publisher, the editors and the reviewers. Any product that may be evaluated in this article, or claim that may be made by its manufacturer, is not guaranteed or endorsed by the publisher.
